# Evaluating Complex Mixtures in the Zebrafish Embryo by Reconstituting Field Water Samples: A Metal Pollution Case Study

**DOI:** 10.3390/ijms18030539

**Published:** 2017-03-02

**Authors:** Ellen D. G. Michiels, Lucia Vergauwen, An Hagenaars, Erik Fransen, Stefan Van Dongen, Steven J. Van Cruchten, Lieven Bervoets, Dries Knapen

**Affiliations:** 1 Zebrafishlab, Veterinary Physiology and Biochemistry, Department of Veterinary Sciences, University of Antwerp, Universiteitsplein 1, 2610 Wilrijk, Belgium; ellen.michiels@uantwerpen.be (E.D.G.M.); lucia.vergauwen@uantwerpen.be (L.V.); anhagenaars@hotmail.com (A.H.); 2 Systemic Physiological and Ecotoxicological Research (SPHERE), Department of Biology, University of Antwerp, Groenenborgerlaan 171, 2020 Antwerp, Belgium; lieven.bervoets@uantwerpen.be; 3 StatUa Center for Statistics, University of Antwerp, 2000 Antwerp, Belgium; erik.fransen@uantwerpen.be; 4 Evolutionary Ecology, Department of Biology, University of Antwerp, Universiteitsplein 1, 2610 Wilrijk, Belgium; stefan.vandongen@uantwerpen.be; 5 Applied Veterinary Morphology, Department of Veterinary Sciences, University of Antwerp, Universiteitsplein 1, 2610 Wilrijk, Belgium; steven.vancruchten@uantwerpen.be

**Keywords:** metals, mixture toxicity, aquatic toxicology, zebrafish embryo, field-to-lab testing

## Abstract

Accurately assessing the toxicity of complex, environmentally relevant mixtures remains an important challenge in ecotoxicology. The goal was to identify biological effects after exposure to environmental water samples and to determine whether the observed effects could be explained by the waterborne metal mixture found in the samples. Zebrafish embryos were exposed to water samples of five different sites originating from two Flemish (Mol and Olen, Belgium) metal contaminated streams: “Scheppelijke Nete” (SN) and “Kneutersloop” (K), and a ditch (D), which is the contamination source of SN. Trace metal concentrations, and Na, K, Mg and Ca concentrations were measured using ICP-MS and were used to reconstitute site-specific water samples. We assessed whether the effects that were observed after exposure to environmental samples could be explained by metal mixture toxicity under standardized laboratory conditions. Exposure to “D” or “reconstituted D” water caused 100% mortality. SN and reconstituted SN water caused similar effects on hatching, swim bladder inflation, growth and swimming activity. A canonical discriminant analysis confirmed a high similarity between both exposure scenarios, indicating that the observed toxicity was indeed primarily caused by metals. The applied workflow could be a valuable approach to evaluate mixture toxicity that limits time and costs while maintaining biological relevance.

## 1. Introduction

In most aquatic ecosystems, organisms are exposed to multiple contaminants of different chemical groups and are therefore continuously exposed to mixtures rather than to single compounds [1]. While environmental monitoring and risk assessment are mainly focused on analysis and effect assessment of single compounds [2], river water and/or sediment can cause toxicity to organisms, even if the individual concentrations of the contaminants are very low (e.g., below the surface water quality standards or below the no observed effect concentrations (NOECs)) [3,4,5]. On the other hand, cases have been reported in which the toxicity of a complex mixture was lower than that predicted based on single compound toxicity [6,7]. Because of the complexity and uncertainties related to mixture toxicity, it is difficult to develop mitigation programs and to improve the quality of rivers, or aquatic ecosystems in general. Instead of aiming to decrease the concentrations of all detected compounds in a contaminated stream, it may be more feasible and cost-efficient to identify the toxicants in the mixture that are predominantly responsible for the observed toxic effects, allowing measures to be taken that specifically address those contaminants [8].

The measurement of selected compounds and evaluation of mixture effects is an approach that is used often. For example, Whitehead et al. [9] used a combination of field-caged experiments and laboratory exposures to field water while Johnson et al. [10] exposed fish in artificial pools up- and downstream of an effluent discharge point. Schoenaers et al. [11] were able to discriminate between different complex field water samples reflecting different types of pollution by using a combination of molecular, biochemical and physiological biomarkers. Several approaches combining bioassays and chemical analyses to identify toxicants of concern have been developed, such as effect-directed analysis (EDA) and Toxicity Identification Evaluation (TIE). Using such approaches, toxicity can then be traced back to single chemicals or to a fraction of the initial mixture [8,12,13,14,15,16,17,18]. EDA and TIE have the same goal, i.e., identifying toxicants of concern, but they apply different methodologies to achieve that goal. A TIE starts with a toxicity test of the original sample using whole organisms. After this initial test, a TIE is divided into three phases. Phase 1 consists of the characterization of the type of contaminants causing toxicity, e.g., metals. This is done by comparing the whole organism toxicity of a sample before and after treatment with ethylenediaminetetraacetic acid (EDTA), aeration, pH adjustment, etc. The second phase involves the identification of the specific toxicants using analytical measurements and the third phase confirms the identified toxicants [15,16,17]. Different techniques can be applied during this third phase (e.g., spiking, deletion, etc.) [17]. An EDA also starts with toxicity tests of the original sample, but these tests are mainly based on in vitro endpoints. Subsequently, sample treatments such as extraction and fractionation are performed, followed by another round of in vitro tests to determine which fraction causes the observed toxicity. These assays are then followed by chemical analyses and finally also by a confirmation step [18].

**Figure 1:**
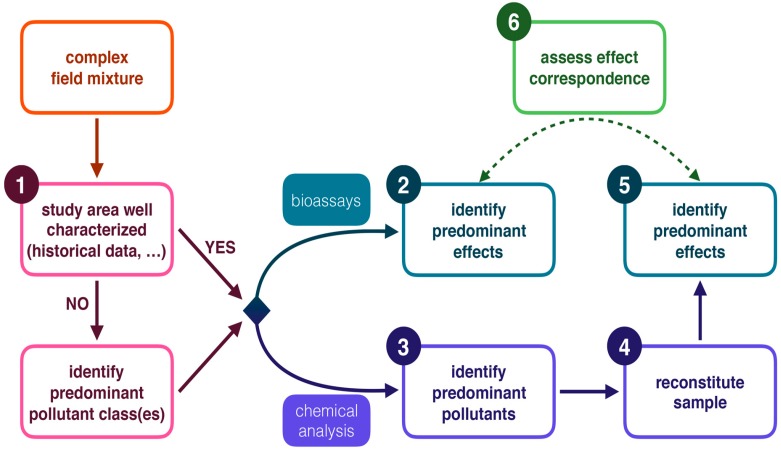
Schematic overview of the workflow of this study analogous to the concepts of a Toxicity Identification Evaluation (TIE). The numbered boxes represent the steps that were conducted in this study. Identification of predominant pollutant class(es) corresponds to phase 1 of a TIE and is necessary in cases where no background data are available (e.g., as illustrated by Burgess et al. [18]). The dashed arrow represents step 6 of the workflow, i.e., comparing the effects between field and reconstituted exposure scenarios.

However, in many cases, knowledge about the presence of specific contaminants is available (e.g., it can be deduced from close-by industrial activities, or derived from documented pollution history) and the role of certain toxicants in a water body can be plausibly expected. In such cases, it may not be necessary to follow the entire workflow of TIE or EDA approaches. We applied a workflow (Figure 1) in which first the toxicity of the complex field sample was determined in vivo (step 1 in Figure 1). Secondly, concentrations of the suspected contaminants were quantitatively measured. Next, the field samples were reconstituted in terms of general physico-chemical properties and by adding the suspected toxicants in the appropriate concentrations. In the fourth step, the toxicity of these reconstituted samples was determined, and, finally, the results were compared to the toxicity of the original complex field sample. The last three steps of our workflow are conceptually analogous to the confirmation phase of a TIE and can possibly be used as an alternative approach. If no historical data are available, phases 1 and 2 of a TIE or a similar approach should be used as a guidance to select candidate toxicants [15,16]. Because these reconstituted samples may more closely resemble the original field samples when compared to samples obtained after fractionation, this approach may also be used to validate results from effect-directed analysis.

Our specific implementation of the overall approach outlined above considered whether morphological and physiological effects in zebrafish embryos, caused by exposure to contaminated environmental water samples, were mainly due to the known predominant contaminants (metals) in selected rivers. For this case study, we selected two Flemish rivers (Scheppelijke Nete, abbreviated as SN, and Kneutersloop, abbreviated as K), and a ditch which flows into SN with a well-known history of metal contamination. Since the toxicity of complex aquatic mixtures to organisms depends on the interaction between chemicals and physicochemical properties of the water (e.g., pH), the test organism used for evaluating toxicity should preferentially be tolerant to a wide range of physicochemical properties. The homeostatic salinity range of zebrafish embryos was therefore established in a preliminary experiment. Zebrafish embryos were then exposed in the lab to water samples taken in the field. Metal concentrations were measured in these samples to identify the concentration of four major cations (Na, Ca, Mg and K) and predominant trace metals. Eight trace metals were identified that exceeded their respective surface water quality standard in at least one of the sample sites (Al, Mn, Fe, Ni, Cu, Zn, As, Cd; see Table 1). Field samples were then reconstituted using the site-specific measured concentrations of the four major cations to reflect the baseline ionic composition of the field samples, and using the site-specific measured concentrations of the eight selected trace metals. Zebrafish embryos were then exposed to the reconstituted mixtures. By comparing effects between exposure to reconstituted and field water samples, we investigated whether the observed toxicity was mainly caused by the metal mixtures rather than by other, unknown, contaminants (e.g., pesticides) or environmental factors. 

## 2. Results

### 2.1. Physicochemical Properties of the Scheppelijke Nete (SN), Ditch (D) and Kneutersloop (K)

Concentrations of Al, Ni, Cu, Zn, As and Cd were higher than the water quality standards for at least one sampling point (underlined in Table 1). Currently, there are no surface water quality standards available for Mn and Fe, but their concentrations were also high in some of the sampling points compared to other rivers and were therefore also included for the reconstitution of the samples [19,20,21]. Apart from Cr and Pb, which were not included in the reconstituted samples and will not be further discussed, the highest metal (loid) concentrations were found in the water of D, and the values were, except for Cu, always higher at the SN2 sampling point than at SN1. Nickel and Cu were the only two elements that were substantially higher in K than in SN. Chromium and Pb never exceeded their surface water quality standards.

The results of the pH, conductivity and measurements of four major cations are listed in Table 2. The pH-values of all sampling points except D were similar to standard embryo medium (pH 7.5). The conductivity in SN was similar to the conductivity of standard embryo medium (500 μS/cm). The conductivities of D and K were higher (931 µS/cm and 1343 µS/cm, respectively) than standard embryo medium (Table 2). 

### 2.2. Exposure to Field Water Samples and Reconstituted Water Samples

#### 2.2.1. Survival

**Figure 2:**
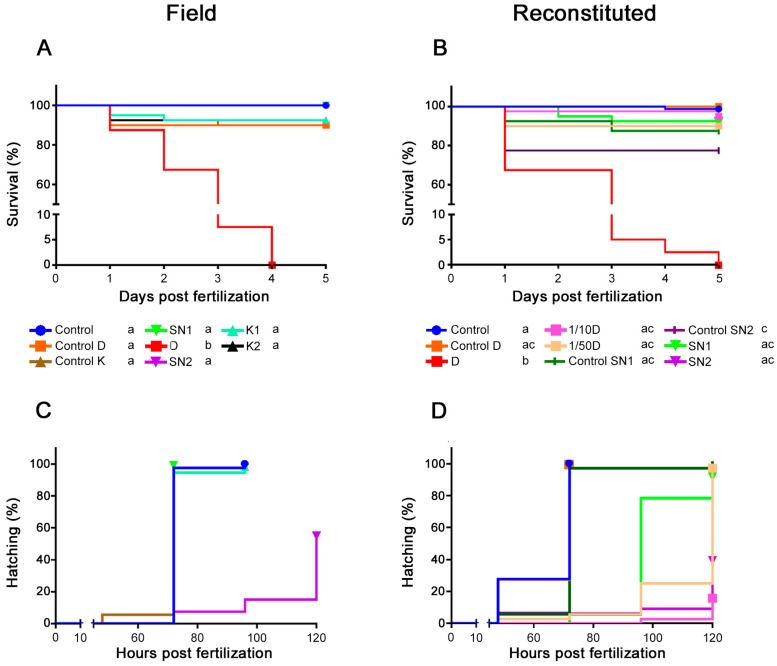
Survival curves after exposure to (**A**) field water samples, (**B**) reconstituted metal mixtures. Hatching curves after exposure to (**C**) field water samples and (**D**) reconstituted metal mixtures. Different small letters in the legend indicate significant differences within each graph. “Control” indicates rearing in standard embryo medium. In (**A**,**C**) separate controls (“control D” and “control K”), with pH and conductivity adjusted to those of the field water samples, were included. The pH and conductivity of the Scheppelijke Nete (SN) resembled standard embryo medium; therefore, a separate control was not needed in this case. In (**B**,**D**), “control D”, “control SN1” and “control SN2” media were reconstituted based on the actual baseline ionic composition (Na, K, Ca and Mg) measured in the field water samples.

All embryos exposed to water collected at D or exposed to reconstituted D died within four days (Figure 2A,B). The diluted D mixtures showed no significant mortality compared to reconstituted control D. A significant number of embryos that were exposed to reconstituted control medium of SN2 (i.e., without the presence of trace metals) died in the first 24 h, but when the same medium was used to produce the metal mixture of SN2, no significant mortality was observed. The exposure of embryos to reconstituted control medium of SN2 was repeated six times and resulted in a high mortality rate in the majority of the experiments (at least 23% mortality in four out of six experiments). Based on our current data, we cannot explain this observation. The positive control of 4 mg/L 3,4 dichloroaniline (DCA) always resulted in an expected mortality of approximately 100% at the highest concentration, which is in compliance with the criteria of Organisation for Economic Co-operation and Development Test Guideline (OECD TG) 236 [22]. The other DCA concentrations (0.5; 1 and 2 mg/L) resulted in 0%, 6% and 17% mortality, respectively. The internal negative controls showed no mortality.

#### 2.2.2. Hatching

Embryos that were exposed to the environmental SN2 water sample (Figure 2C) or to the reconstituted mixture of SN2 (Figure 2D) showed a significant (*p* < 0.001) decrease in hatching success at 120 h post fertilization (hpf) compared to the controls (45% and 61% of the embryos did not hatch, respectively) and a hatching delay. The hatching effect of SN2 and reconstituted SN2 was comparable (*p* = 0.18). The embryos exposed to the reconstituted dilutions of D had hatching problems as well compared to the embryos exposed to reconstituted control D, with the highest percentage of unhatched embryos (84%) being recorded in the 1/10D group. Embryos exposed to reconstituted SN1 also showed a hatching delay (compared to reconstituted control SN1), while the embryos exposed to environmental SN1 (compared to standard embryo medium (=control) embryos) did not. All embryos that were not exposed to metals hatched at a normal hatching rate (i.e., hatching occurred between 48 and 72 hpf).

#### 2.2.3. Length

**Figure 3:**
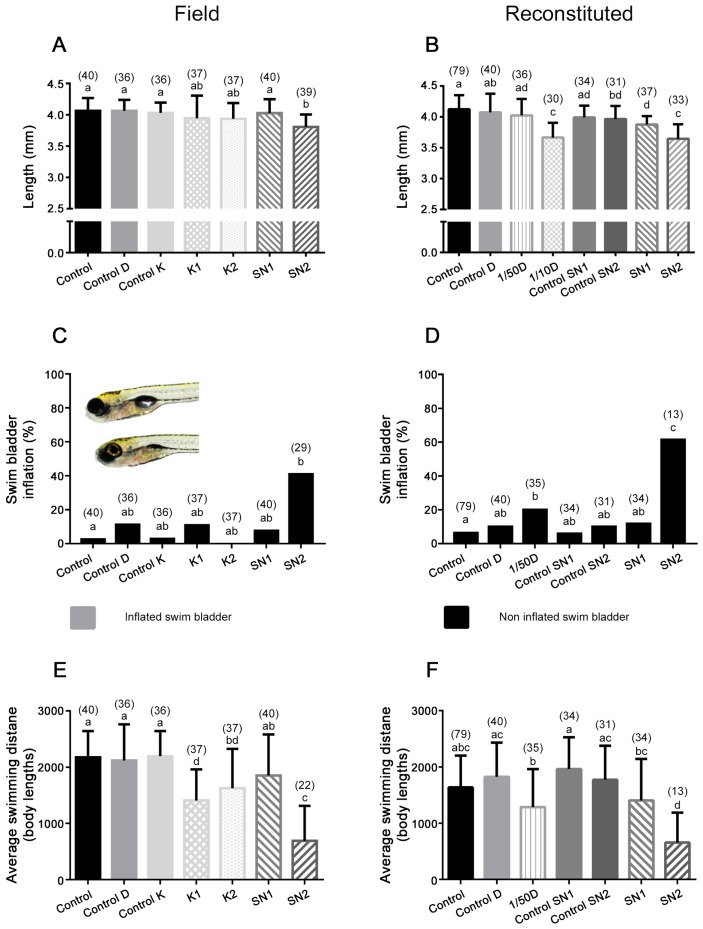
Sub-lethal effects after exposure to field water samples or to reconstituted metal mixtures. Length of the larvae (**A**) exposed to field water samples or (**B**) to reconstituted metal mixtures. Percentages of swim bladder inflation of all hatched larvae at 120 hpf (**C**) after exposure to field water samples, and (**D**) after exposure to reconstituted mixtures. Representative photograph: the upper larva has an inflated swim bladder and the bottom one has a non-inflated swim bladder. Average swimming distance of (**E**) larvae exposed to field water samples, and (**F**) to reconstituted metal mixtures. For an explanation of the controls, see Figure 2. Different small letters indicate significant differences (*p* < 0.05) (*n* is given in parentheses).

Only the group that was exposed to water of SN2 had a smaller (*p* ≤ 0.001; for the full ANOVA summary, see Table S1) body length (3.8 ± 0.2 mm) compared to the control (4.1 ± 0.2 mm) (Figure 3A). In the experiment with the reconstituted solutions, 1/10D and SN2 (i.e., the groups with the highest metal concentrations) had the smallest body lengths (3.7 ± 0.2 and 3.6 ± 0.2 mm, respectively). The larvae exposed to reconstituted SN1 also had significantly (*p* ≤ 0.001; for the full ANOVA summary, see Table S1) smaller body lengths (3.9 ± 0.1 mm) compared to the control larvae (4.1 ± 0.2 mm) (Figure 3B).

#### 2.2.4. Morphological Abnormalities

None of the percentages of morphological abnormalities were significantly different from controls, except for impaired inflation of the posterior chamber of the swim bladder at 120 hpf (Figure 3C,D). A significant percentage of embryos exposed to environmental SN2 (41%) compared to standard embryo medium (=control) embryos had a non-inflated swim bladder. Embryos exposed to reconstituted SN2 (62%) also showed impaired swim bladder inflation compared to reconstituted control SN2 embryos. In the 1/50D exposure, there was also a significant increase of embryos with a non-inflated swim bladder (20%) compared to reconstituted control D. The percentage of control embryos with a non-inflated swim bladder never exceeded 11%. As swim bladder inflation was only scored when embryos successfully hatched, and hatching was unsuccessful after exposure to 1/10D, these results are not shown in Figure 3C,D.

#### 2.2.5. Swimming Activity

The swimming distance travelled in 50 min (expressed as body lengths (BL)) was decreased (*p* ≤ 0.01; for the full ANOVA summary, see Table S1) for embryos exposed to water from K1 and K2, compared to control K. The distance travelled by embryos exposed to reconstituted SN2 water (657 ± 534 BL; average ± standard deviation) was similar to the SN2 exposed embryos (690 ± 624 BL) and significantly (*p* ≤ 0.001; for the full ANOVA summary, see Table S1) smaller than the controls (standard embryo medium and reconstituted control SN2) (Figure 3E,F). Embryos exposed to 1/50D also showed a decrease (*p* ≤ 0.01; for the full ANOVA summary, see Table S1) (1289 ± 676 BL) compared to the controls of D (1826 ± 606 BL) (Figure 3F). Because almost no embryos exposed to 1/10D hatched, they were not included in the swimming activity analysis. The groups with a decreased swimming distance also show a decreased swimming velocity (supplementary information, Figure S1). 

Based on the results of the morphological scoring, and the knowledge that swim bladder inflation is important for buoyancy and therefore swimming, the swimming data of the embryos with and without inflated swim bladder were compared (supplementary information, Figure S2). These results show that the total distance travelled was the lowest for embryos with a non-inflated swim bladder. However, swimming activity was also significantly lower for the SN2 (*p* ≤ 0.001) and reconstituted SN2 (*p* = 0.004) exposed embryos with an inflated swim bladder compared to the control embryos that had an inflated swim bladder. 

#### 2.2.6. Metal Measurements of the Reconstituted Mixtures

Table 3 presents the trace metal (loid) exposure concentrations in the medium of the reconstituted mixtures (average ± SD). All measurements of reconstituted control media were below the detection limit, except for Ni (Table 3). The detection limit was 0.1 µg/L for every element, except for Al and Fe, which had a detection limit of 1 µg/L. Comparison between the medium before and after renewal showed that all metal concentrations decreased significantly over 48 h (between 6% and 44%), except for Mn and Ni, which remained more or less constant (supplementary information, Figure S3).

#### 2.2.7. Identification of the Similarity between Field and Reconstituted Exposure Scenarios

**Figure 4:**
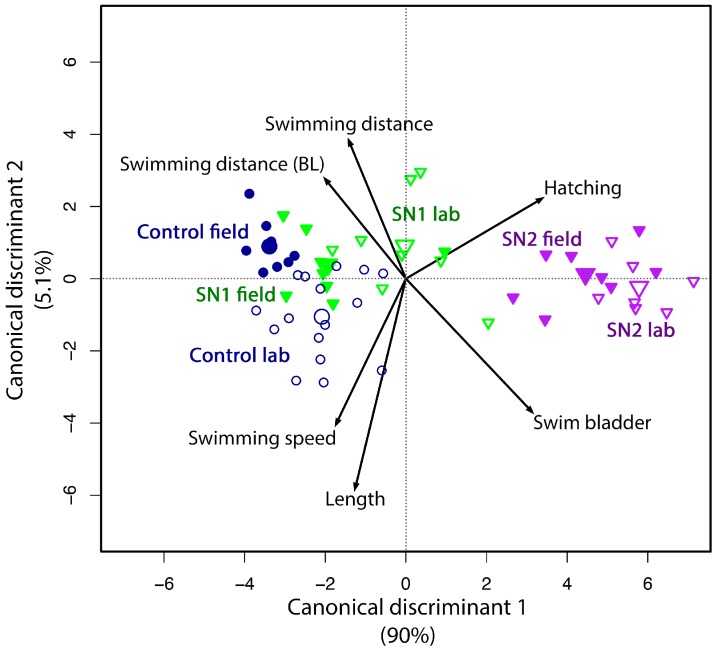
Canonical discriminant analysis of the results of the experiment with field water and the reconstituted mixtures. Small symbols represent the sample scores, and large symbols represent the average scores per experimental group. Filled symbols represent samples of the field experiment and open symbols represent samples of the reconstituted experiment. Control field and control lab represent standard embryo medium. Length, swimming distance and speed are positive effects (i.e., higher values are better), while hatching and swim bladder represent impaired hatching and impaired swim bladder inflation, which are adverse effects.

A canonical discriminant biplot (Figure 4) with both exposure scenarios was constructed. Figure 4 shows the scores for the first two axes. This analysis included all experimental groups that were present in both set-ups (i.e., SN1, SN2 and controls) and the sub-lethal effects to explore how sub-lethal effects differed between experimental groups and to determine the similarity between the field and reconstituted exposure scenarios. For example, the analysis showed that impaired swim bladder inflation and impaired hatching showed similar differences among experimental groups (small angle between the vectors), even though only hatched larvae were scored for swim bladder inflation. The observation that larvae with a non-inflated swim bladder showed a lower swimming activity and that all swimming parameters and larval length were correlated to each other, was also reflected in the graph. The samples of standard embryo medium were positively associated to all swimming parameters and length. As the corresponding experimental groups (control, SN1, SN2) of the field and reconstituted experiment were located in close proximity to each other, this confirmed that the effects of the treatments were comparable for the two scenarios.

## 3. Discussion

Identification of the contaminants which are contributing the most to the observed toxicity remains difficult when dealing with mixture toxicity. In our approach, delineated in Figure 1, a comparison between exposure of zebrafish embryos to field samples and reconstituted samples was used to confirm that this specific combination of metals was sufficient to explain the observed toxic effects.

### 3.1. Contamination History of the Study Area

Scheppelijke Nete (SN) and Kneutersloop (K), both part of the Scheldt drainage basin in Flanders (Belgium), were used as a case-study to evaluate the applied workflow (Figure 1) because of their well characterized metal contamination history. The concentrations of Cd, Cr, Cu and Ni in SN that were measured in our study were slightly lower or in the same range compared to another study (2003) in which rivers of the same tributary (Grote Nete) of the Scheldt were studied, but Pb was approximately twice as high for every sampling point [23]. This suggests that the overall water quality of SN was stable for most trace metals over the last decade. The concentrations of Cd, Ni and Zn were higher in the ditch (D) compared to all other sampling points. This was also the case in the study of Bervoets and Blust [23]. At the SN2 sampling point, positioned downstream of the conjunction of D with SN, higher concentrations of most metals were measured, compared to the SN1 sampling point. This confirms that D is an important contamination source of SN. Copper and Ni were abundant in K, but concentrations were seven to 40 times lower than in 2004 [24] and three to nine times lower than in 2010 [25]. The water quality of K therefore appears to have improved, although the concentrations of Cu and Ni were still eight (Cu) and 1.5 (Ni) times above the surface water quality standards. Using this large amount of historical data, we directly performed targeted analytical measurements without first characterizing the presence of specific classes of contaminants, which is normally done in the first phase of a TIE [15]. We then assessed whether this information was sufficient to explain the observed toxicity.

### 3.2. Comparison between the Toxicity of Field and Reconstituted Water Samples

#### 3.2.1. Toxicity in the Selected Streams Was Mainly Caused by the Metal Mixtures

Both the single morphological and physiological endpoints, as well as the canonical discriminant analysis, show a remarkable similarity between the toxicity observed after direct exposure to the field samples and the reconstituted metal mixtures. In Figure 4, the samples of the field experiment and their reconstituted counterpart showed relatively low differences in the toxicity effects compared to differences among the different sampling points. The negative effects (e.g., impaired hatching and impaired swim bladder inflation) were associated with SN2, which is the sampling point downstream of the source of pollution. On the other hand, SN1 samples were positioned closely to the control samples (standard embryo medium), which were associated with the positive endpoints (length and the measures of swimming activity). This indicates that SN1, which is located upstream of the pollution source, was indeed less toxic than SN2. Overall, these results indicate that the observed toxicity in the selected streams was mainly caused by the metal mixtures rather than by other, unknown, contaminants (e.g., pesticides) or environmental factors.

#### 3.2.2. More Detailed Toxicity Analysis of the Selected Sites

The most distinct effect was that the water of D caused 100% mortality in both experiments (Figure 2), which is caused by the extremely high metal concentrations (Table 1). When hatching of the embryos in field and reconstituted water is compared, it is clear that SN2 exposed embryos were affected in both waters in a similar way (*p* = 0.18 for the comparison between SN2 and reconstituted SN2). However, embryos exposed to SN1 water (upstream of the pollution source) were affected in the reconstituted experiment (Figure 2) but not after exposure to field water samples. Embryos exposed to reconstituted SN1 were also smaller, while SN1 exposed embryos were not. There are various possible reasons for the slightly higher toxicity in the reconstituted samples (which is also visible in Figure 4), e.g., differences in the bioavailability of the trace metals, unknown contaminants with antagonistic effects in the field water samples, influence of organic materials in the field water samples, etc.

A decreased hatching success and/or hatching delay after exposure of zebrafish to Cd, Mn or Zn has already been described in several papers [26,27]. This has been hypothesized to be caused by an inhibition of the proteolytic function of the hatching enzyme [26]. The highest values for these three metals are found in the SN2 and D dilution sampling points (Table 1), and the embryos exposed to those mixtures also have the highest effect on hatching. The fact that hatching and swim bladder inflation are positioned in the same direction in the canonical discriminant analysis (Figure 4) could be due to the delayed hatching, which prevents swim bladder inflation before 120 hpf. On the other hand, 44% of the larvae of the SN2 group and 40% of the reconstituted SN2 embryos were not hatched at 96 hpf, but were hatched and did have an inflated swim bladder at 120 hpf. The effect of delayed hatching on swim bladder inflation is therefore not always straightforward to predict. Impaired swim bladder inflation can also be caused by altered metabolism, for example disturbing gas passage to the swim bladder [28], but because swim bladder inflation is dependent on a combination of different processes [29], it is difficult to determine which mechanism is primarily involved after metal exposure without further research. Fe has been shown to stimulate hatching in zebrafish at a pH between 7 and 7.5 [30], which is opposite to our findings based on the metal measurements (Table 1). This suggests that the other metals may have an antagonistic effect.

## 4. Materials and Methods 

### 4.1. Fish Maintenance and Egg Production 

According to EU Directive 2010/63/EU and the Commission Implementing Decision 2012/707/EU, fish are non-protected animals until the stage of free feeding. This limit was set at 120 hpf for zebrafish. Experiments of this study did not exceed 120 hpf, but were nevertheless part of a larger project for which approval by the Ethical Committee for Animals of the University of Antwerp was obtained (project number 2011-05, 1 December 2011). Fish husbandry and all experiments were carried out in strict accordance with the EU Directive on the protection of animals used for scientific purposes (2010/63/EU). Adult wild type zebrafish (*Danio rerio*) were obtained from an in house zebrafish line and kept in a rack with recirculating standard embryo medium (ZebTec standalone, Tecniplast, Buguggiate, Italy) in a temperature controlled room at a 14/10 h light/dark cycle. Standard embryo medium was made with reverse osmosis water to which Instant Ocean^®^ salts (Blacksburg, VA, USA) and NaHCO_3_ were added until a conductivity of 500 µS/cm and a pH of 7.5 were reached. The rack was continuously filtered by a mechanical, biological and UV filter. The water temperature was set to 28 ± 0.2 °C, the pH at 7.5 ± 0.3 and the conductivity at 500 ± 15 µS/cm, with 35% of the circulating water being renewed daily. The ammonium, nitrate and nitrite concentrations were measured twice a week using Tetra tests (Tetra, Melle, Germany). The values were always below 0.25 mg/L for ammonium, below 0.3 mg/L for nitrite and the concentrations for nitrate did not exceed 25 mg/L. Fish were fed 4 times per day: 2 times 1.5% of their mean wet weight of granulated food (Biogran medium, Prodac International, Cittadella, Italy) and 2 times with frozen food (*Artemia nauplii*, *Daphnia*, *Chironomidae* larvae or *Chaoborus* larvae). Breeding tanks inside the rack were used for reproduction. Fish were placed inside the breeding tanks in the evening, in a ratio of 1 male/2 females. Males and females were separated overnight, and the divider was removed in the morning. Spawning and fertilization occurred immediately after the lights were switched on. After 30 to 40 min, the eggs were collected and faeces and other impurities were removed. The eggs were placed in standard embryo medium or test solutions within 2 hpf and checked for fertilization (cell division) and cleanness of the chorion.

### 4.2. Study Area

**Figure 5:**
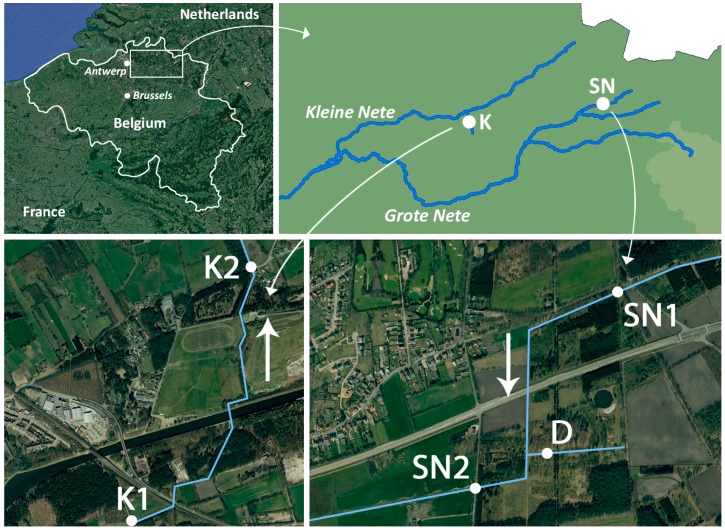
Map of the sampling sites. The Scheppelijke Nete is indicated as SN, the ditch as D and the Kneutersloop as K. The arrows indicate the direction of the flow.

The Scheppelijke Nete (SN) and the Kneutersloop (K) are small streams that are both part of the Scheldt drainage basin (Belgium, Figure 5). They are known for their historical metal contamination [25,31]. Three different sampling points were selected in SN and two sampling points in K (Figure 5). A ditch (D), which is located between SN1 and SN2 (Figure 5), is the contamination source of SN. SN1 is located upstream of D and low contaminant concentrations are expected at this site. The geographic coordinates of all sampling points can be found in supplementary information, Table S2. Field water samples were stored at 4 °C in glass bottles before zebrafish embryo exposure. The pH and conductivity of each water sample (sampling date: 1 July 2014) were measured before filtration (0.2 µm, Whatman 1001-150) of the samples. Aliquots of these filtered water samples were acidified to 1.5% HNO_3_ (69%, 150 µL/10 mL sample, Merck, Darmstadt, Germany) and stored at 4 °C until metal analysis.

### 4.3. General Zebrafish Embryo Exposure Procedure

Embryos were placed in 48-well plates (1 embryo per well), of which 8 wells were filled with standard embryo medium and used as an internal negative control. As a positive control, another plate was filled with 3,4-dichloroaniline (Sigma Aldrich, Diegem, Belgium nominal concentrations 0.5, 1, 2 and 4 mg/L) and 12 embryos were exposed to each concentration [22]. One full negative control plate with standard embryo medium was included. All plates were covered with Parafilm (Bemis Europe, Soignies, Belgium), placed in an incubator (Panasonic MIR-254-PE, Rotselaar, Belgium,) at 28 ± 0.5 °C at a 14/10 h light/dark cycle. A mortality check was performed daily (coagulation, lack of somite formation, non-detachment of the tail, lack of heartbeat [22]). Hatching was checked from 48 hpf onward and empty chorions were removed daily. Unhatched embryos were manually dechorionated at 5 dpf with forceps before a complete scoring of morphological abnormalities was carried out. Dechorionated embryos were not scored for swim bladder inflation since they had not been able to reach the water surface to initiate the inflation process. Non-inflation of the swim bladder was considered a consequence of non-hatching and not a direct effect in these cases. The morphological scoring involved 28 binary parameters (supplementary information, Table S3). Every plate was analyzed for swimming activity at 5 dpf in a tracking device (Zebrabox 3.0, Viewpoint, Lyon, France) for 50 min under normal light conditions (100% light setting, 1160 lux). Travelled distance, duration of activity, rotations and angles were determined (ZebraLab software version 3.20.5.104, Viewpoint, Lyon, France). After the tracking analysis, larvae were anesthetized using 0.1 g/L MS-222 (tricaine methanesulfonate, Sigma-Aldrich, Diegem, Belgium) at pH 7.5 and photographed with a calibrator using a Canon 600D (18 megapixel, Canon, Diegem, Belgium) mounted on a Leica APOS8 stereomicroscope (Leica Microsystems, Diegem, Belgium). Standard length was determined in groups of 5 larvae using Image J software version 1.47v (U.S. National Institutes of Health, Bethesda, MD, USA) with the photographed reference mark as a calibrator. Experiments were only considered valid if (1) ≤10% of the negative controls died; (2) ≥80% of the negative control embryos hatched by the end of the test (5 dpf); and (3) at least 30% mortality was observed at 5 dpf when the embryos were exposed to the highest concentration of the positive control (4 mg/L 3,4-dichloroaniline) [22].

### 4.4. Determination of Salinity Tolerance Range

The salinity tolerance range of zebrafish embryos was determined in a preliminary experiment by exposing embryos from 2 to 120 hpf to 9 different salt concentrations (50, 100, 200, 400, 500, 800, 1600, 3200, 3900 µS/cm, corresponding to 0.025, 0.05, 0.1, 0.2, 0.25, 0.4, 0.8, 1.6, 1.95 g/L salts), which were prepared with Instant Ocean^®^ salts in reverse osmosis water. If necessary, pH was adjusted to 7.5 with NaHCO_3_ solution (30 g/L). In addition to the morphological and physiological scoring, photographs with a calibrator were taken at 24 hpf and the diameter of the chorion was determined using Image J software (1.47v). The diameter of the chorions decreased significantly as a function of conductivity (supplementary information, Figure S4). However, from 400 to 1600 µS/cm, which is the conductivity range of all field water samples, there was no significant effect (*p* = 0.26, for full ANOVA summary see supplementary information, Table S1) on the diameter of the chorion. This provides confidence that the zebrafish embryo can be used for studying direct toxicity of field freshwater samples with different physiochemical properties. Given our data, we do not expect that effects observed after exposure to field or reconstituted water were directly caused by differences in e.g., salinity.

### 4.5. Exposure to Field Water Samples

Embryos (*n* = 40, see Section 4.3) were exposed to the different filtered field water samples (SN1, SN2, D, K1, K2) immediately after sampling to prevent the water composition from changing (e.g., biodegradation, photodegradation, precipitation,…). In addition to standard embryo medium (=control), D and K control media (field control D and field control K) were prepared with conductivity and pH values adjusted to the field sample values using Instant Ocean^®^ salt and HCl or NaOH (for an overview of all control media see Table S4). Because the pH and conductivity of SN were similar to standard embryo medium, no site-specific control was used for SN1 and SN2. The plates were saturated with the different solutions one day in advance, and solutions were renewed prior to the start of the experiment. The media were then renewed every 48 h. A morphological and physiological scoring was performed to assess sub-lethal toxic effects (see Section 4.3) and standard length was determined. Distance travelled was expressed as body lengths (BL) when coupled length-distance data were available (calculated using the mean length of 5 larvae) and as absolute distance in other cases.

### 4.6. Metal Analysis

The environmental water samples were analyzed for 9 trace metals (Al, Cr, Mn, Fe, Ni, Cu, Zn, Cd, Pb), 1 metalloid (As) and 4 essential elements (Na, Ca, K, Mg). All samples were analyzed using a quadrupole inductively coupled plasma mass spectrometer (ICP-MS; Agilent 7700× ICP-MS, Santa Clara, CA, USA). Measuring accuracy was determined by measuring certified Trace Elements in Water solution (SRM 1643e). Recovery of SRM 1643e reference material was within 5% of the certified values.

### 4.7. Exposure to Reconstituted Water Samples

In addition to standard embryo medium (=control), control media (reconstituted freshwater) were prepared to resemble natural water samples taken at each sampling point (reconstituted control D, reconstituted control SN1 and reconstituted control SN2) by using the measured concentrations of Na, K, Mg, Ca in the field water samples. All 4 ions were added as salts (CaCl_2_, MgSO_4_, KCl and NaHCO_3_; VWR, Heverlee, Belgium). These control media were also used as a basis to produce the metal mixture solutions. Metal salts of the trace metal ions and the metalloid (CdCl_2_·2.5H_2_O, ZnCl_2_, MnCl_2_·4H_2_O, FeCl_2_·4H_2_O, NiCl_2_·6H_2_O, CuCl_2_·2H_2_O, AlCl_3_·6H_2_O, Na_2_HAsO_4_·7H_2_O; Sigma-Aldrich, Diegem, Belgium) that exceeded the surface water quality standards (Table 1) in at least one of the sampling sites were used to produce the reconstituted metal mixtures. HCl and NaOH were used to adjust the control and test solutions to the pH that was measured in the field (see Section 4.2). Positive, negative and internal controls were implemented as described previously (see Section 4.3). Because almost no malformations were seen in embryos exposed to K1 or K2 field water, no reconstituted mixtures were made for this river. Since embryonic mortality was 100% after exposure to D field water, two dilutions of the reconstituted D mixture (1/10 and 1/50) were prepared. All plates were saturated in advance. To prevent an osmotic shock and to allow physiological acclimation to different water compositions, all embryos, control and exposed ones, were first placed in a 20% solution of the respective reconstituted control medium diluted with standard embryo medium for two hours. Metals were added to this solution for the exposures. The percentage of the reconstituted control medium was then gradually increased by 20% every two hours until 100% was reached. During the exposure experiment, replicated water samples were taken before and after the medium was renewed. These samples were stored at −20 °C and thawed the evening before analysis, after which they were acidified to 1.5% HNO_3_. At least 2 replicated samples were measured for each condition.

### 4.8. Statistical Analysis

All statistical tests were performed using GraphPad Prism 6 (GraphPad Software, San Diego, CA, USA), SPSS Statistics 22 (IBM, Armonk, NY, USA) or R (version 3.1.2, R Foundation for Statistical Computing, Vienna, Austria). All graphs were made using GraphPad Prism 6 (GraphPad Software, San Diego, CA, USA), except for the graph of the canonical discriminant analysis which was made in R. Next to all univariate analyses, we also explored differences in biological effects between the different groups multivariately. The aim of this approach is to provide a graphical overview of the similarities in the effects of the field experiment groups and their reconstituted counterparts. D and D1/10 samples were not included in the canonical discriminant analysis because embryos died or had mostly not hatched, resulting in missing values for many variables. On the basis of a canonical discriminant analysis of the 6 groups, a plot of the canonical means, factor loadings and sample scores was produced. In this way, all information available is approximated in a single two-dimensional plot. The cut off for statistical significance was set to 0.05. For analysis of survival and hatching curves, pairwise logrank tests were carried out and a Bonferroni correction was applied. The mean decline in diameter of the chorion per unit change in concentration was estimated by linear regression. Subsequently, we tested whether conductivity significantly affected the diameter of the chorion in the range from 400 to 1600 µS/cm (i.e., the range of the collected field samples) with one-way ANOVA followed by Tukey’s multiple comparisons test. Swimming activity and length were normally distributed and compared between treatment groups using one-way ANOVA. Since malformations were scored using a binary scale, binary logistic regression models were fitted and odds ratios estimated (using MedCalc, Ostend, Belgium [32]). In the case of a “zero cell count” (an event that occurs when all larvae have the same score), odds ratios were estimated by adding a small number (0.5) to each cell [33]. Paired student’s *t*-tests were used to compare medium samples before and after water renewal.

## 5. Conclusions 

Both morphological (survival, hatching, swim bladder inflation, length) as well as physiological parameters (swimming activity) showed a remarkable similarity between the effects observed in the experiments with environmental water samples and reconstituted metal mixtures. The magnitude of the effects was comparable between the experiments as well. A canonical discriminant analysis further indicated that the field experiment groups showed highly similar toxicity effects compared to their reconstituted counterparts. We can therefore conclude that the effects of the environmental water samples in our case study were indeed primarily caused by metal mixture toxicity rather than by other unknown toxicants. 

In addition, assessing the toxic effects of field water samples (step 2, Figure 1) by evaluating morphological as well as physiological endpoints in zebrafish embryos was demonstrated to be a valuable approach. The embryos were sensitive enough to develop malformations and/or show altered swimming activity in several exposure groups. In addition, the overall effect profiles were different among streams, but the toxicity of the reconstituted mixtures was highly similar compared to the toxicity of the field water samples. Both the malformations as well as the magnitude of the effects matched. Our results show the potential of this approach for characterizing complex mixtures. The small discrepancies that were observed between effects after exposure to field and reconstituted samples could possibly be further explained when additional factors (e.g., other pollutants, additional physicochemical properties, etc.) would be considered in addition to the metal mixture used in this study.

It is clear, however, that at least some prior knowledge on the type of contamination is necessary to be able to immediately carry out targeted analytical measurements. This study design could therefore be especially valuable to effluent testing since the predominant contaminants are often known for many types of effluents. On the other hand, a significant discrepancy between effects observed after exposure to field versus reconstituted samples could serve as a biologically relevant indicator for the presence of other previously undetected contaminants, leading to additional efforts to analyze the industrial history of specific sites and to further characterize the chemical composition until a mixture description has been reached that explains all observed biological effects. Finally, also for other applications, such as studies where caged fish are exposed in the field (e.g., [11]), the proposed workflow using zebrafish embryos could be a valuable alternative to reduce both the use of animals and the cost while maintaining biological relevance.

## Figures and Tables

**Table 1:** Dissolved trace metal(loid) concentrations at the different sites. The underlined metal concentrations exceeded the surface water quality standards. Elements shaded in grey were used to reconstitute the metal mixtures. All values are in µg/L.

(µg/L)	Al	Cr	Mn	Fe	Ni	Cu	Zn	As	Cd	Pb
SN1	53.0	0.4	31.1	267	2.4	12.5	65.8	2.5	0.5	2.9
D	904	0.3	5298	10440	67.2	3.0	98621	131	3383	2.2
SN2	84.3	0.3	264	709	5.0	4.3	3447	2.9	6.7	2.1
K1	12.6	0.3	26.1	847	34.9	60.4	46.2	7.8	1.4	3.3
K2	10.4	0.4	17.7	847	35.4	56.4	27.4	6.3	0.9	2.9
Norm	87 ^a^	5 ^b^	/ ^c^	/ ^c^	20 ^b^	7 ^b^	20 ^b^	3 ^b^	0.08–0.25 ^b^ (*)	7.2 ^b^

* Surface water quality standard for Cd is dependent on hardness (0.08 < 40 mg CaCO_3_/L; 0.25 ≥ 200 mg CaCO_3_/L). ^a^ The surface water quality standard for Al was obtained from US Environmental Protection Agency (EPA) standards because there is no Belgian or European surface water quality standard for this element; ^b^ The surface water quality standards were obtained from the Flemish environmental legislation (VLAREM), which is based on the European legislation (EU 2008/105/EG); ^c^ Currently (2016), no surface water quality standards for Mn and Fe exist in the EU or the US.

**Table 2:** Measurements of pH, conductivity and the concentration of four major cations in the field water samples and standard embryo medium.

Sample	Ca (mg/L)	Mg (mg/L)	K (mg/L)	Na (mg/L)	pH	Conductivity (µS/cm)
SN1	54.05	6.50	9.01	28.89	7.47	476
D	51.16	13.60	12.92	39.22	6.14	931
SN2	54.31	6.82	8.46	28.57	7.39	509
K1	38.16	8.36	8.78	208.24	7.33	1343
K2	37.61	8.26	8.50	201.74	7.42	1322
Standard embryo medium	2.86	8.56	5.08	68.94	7.50	500

**Table 3:** Average metal (loid) concentrations ± standard deviation (SD) after addition of the reconstituted mixtures to the wells. BDL = below detection limit. Standard embryo medium was used as a negative and internal control. Sampling points are in the same sequence as the contamination gradient.

µg/L (% of Nominal)	Al	Mn	Fe	Ni	Cu	Zn	As	Cd
SN1 (*n* = 3)	52 ± 0 (98.4%)	30.9 ± 0.4 (99.1%)	253 ± 5 (95.1%)	2.6 ± 0.09 (109.9%)	12.3 ± 0.2 (97.8%)	64.3 ± 1.1 (97.7%)	2.4 ± 0.09 (92.8%)	0.6 ± 0.04 (107.2%)
D (*n* = 2)	926 ± 10 (102.4%)	5446 ± 50.2 (102.8%)	10582 ± 842 (101.4%)	64.6 ± 0.02 (96.1%)	2.9 ± 0.3 (98.3%)	98550 ± 1089 (99.9%)	128 ± 0.6 (97.9%)	3290 ± 58.7 (97.2%)
1/10D (*n* = 3)	93 ± 1 (102.3%)	523 ± 3.2 (98.7%)	1069 ± 49 (102.4%)	6.9 ± 0.09 (102.9%)	BDL	9691 ± 84.1 (98.3%)	12.8 ± 0.08 (98.0%)	342 ± 7.8 (101.2%)
1/50D (*n* = 3)	18 ± 0 (99.6%)	98.8 ± 2.3 (93.3%)	196 ± 14 (93.8%)	1.5 ± 0.2 (109.9%)	BDL	1854 ± 52.1 (94.0%)	2.6 ± 0.07 (101.2%)	63.9 ± 2.5 (94.4%)
SN2 (*n* = 3)	82 ± 1 (97.6%)	257 ± 1.7 (97.5%)	672 ± 7 (94.7%)	4.8 ± 0.1 (97.1%)	4.8 ± 0.2 (112.6%)	3256 ± 27.1 (94.4%)	2.7 ± 0.04 (94.1%)	6.9 ± 0.08 (102.7%)
Standard embryo medium	BDL	BDL	10	0.1	1.3	5.1	BDL	BDL
Control SN1	BDL	BDL	BDL	0.9	BDL	BDL	BDL	BDL
Control D	BDL	BDL	BDL	0.5	BDL	BDL	BDL	BDL
Control SN2	BDL	BDL	BDL	0.6	BDL	BDL	BDL	BDL

## Supplementary Materials

Supplementary materials can be found at www.mdpi.com/1422-0067/18/3/539/s1.
