# Supplementary material for: Evaluating Complex Mixtures in the Zebrafish Embryo by Reconstituting Field Water Samples: A Metal Pollution Case Study
Source: Int J Mol Sci. 2017 Mar 2;18(3):539. doi: 10.3390/ijms18030539 (PMC5372555; doi:10.3390/ijms18030539)
Supplement: Supplementary file 1 [file ijms-18-00539-s001.pdf]

Supplementary Materials: Evaluating Complex Mixtures in the Zebrafish Embryo by Reconstituting Field Water Samples: A Metal Pollution Case Study

Ellen D. G. Michiels, Lucia Vergauwen, An Hagenaars, Erik Fransen, Stefan Van Dongen, Steven J. Van Cruchten, Lieven Bervoets and Dries Knapen

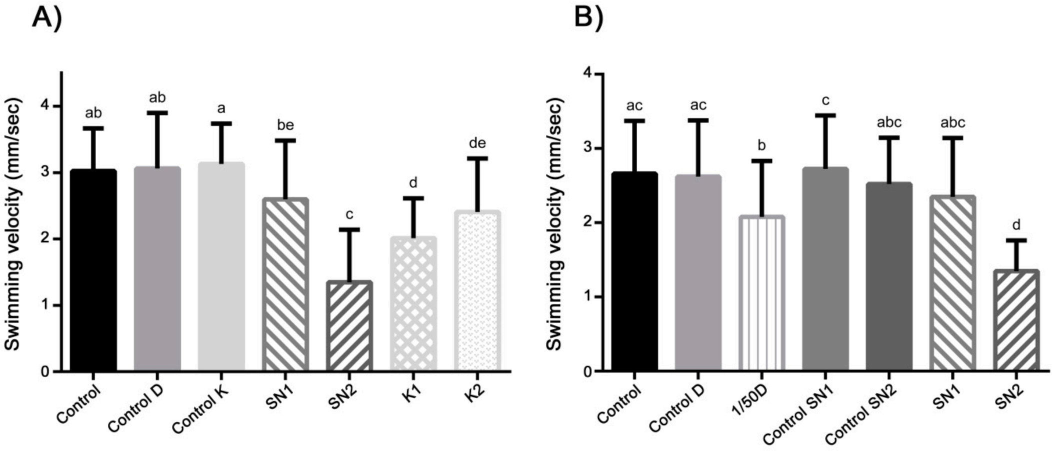

Figure S1. Swimming velocity at 120 hpf of the hatched larvae exposed to environmental water samples (**A**) and reconstituted metal mixtures (**B**). Swimming velocity was calculated for each hatched embryo separately. Letters indicate significant differences.

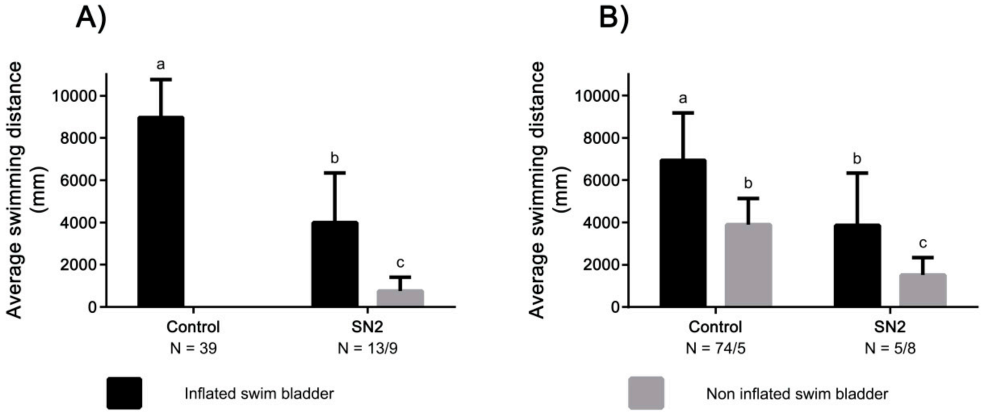

Figure S2. Comparison of average swimming distance between larvae with and without swim bladder inflation after exposure to field samples (**A**) and reconstituted mixtures (**B**). Letters indicate significant differences.

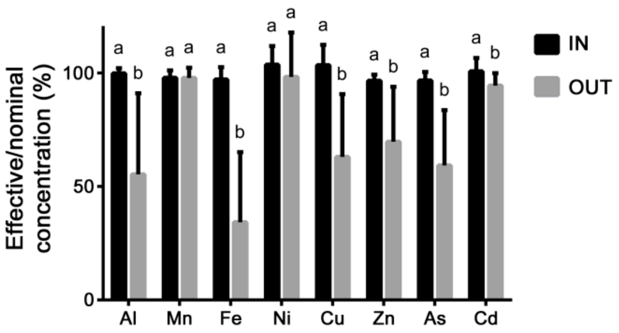

Figure S3. Average percentages of nominal metal concentrations (+SD) in medium samples before and after medium renewal (n =14 for IN samples and n = 13 for OUT samples for each metal). Percentages were calculated as the quantified concentration divided by the nominal concentration. Significant differences were calculated between IN and OUT concentrations for each metal (indicated by letters above bars).

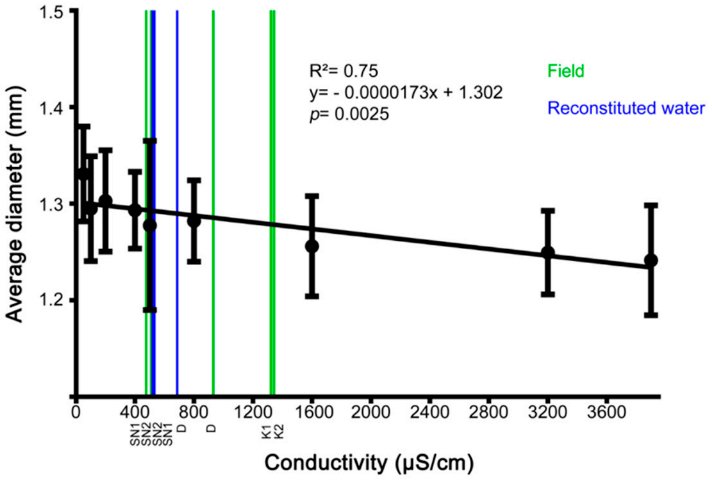

Figure S4. Conductivity dependence of the average diameter of the chorion at 24 hpf. Vertical lines show conductivity values of field water samples and reconstituted water samples.

Table S1. Full One-way ANOVA summary.

	p-value	F	DFn, DFd
Diameter of the chorion	0.26	1.37	3, 70
Length (field water)	≤ 0.001	6.06	6, 258
Length (reconstituted water)	≤ 0.001	16.14	14, 531
Swimming distance (field water)	≤ 0.01	22.55	6, 241
Swimming distance (reconstituted water)	≤ 0.01	9.98	12, 425

Table S2. Coordinates of all sampling points.

Code	River	Basin	Location	Coordinates	
SN1	Scheppelijke Nete	Nete/Scheldt	Mol	51° 12′ 38.20″ N	5° 12′ 51.50″ E
SN2	Scheppelijke Nete	Nete/Scheldt	Mol	51° 12′ 16.04″ N	5° 12′ 25.59″ E
D	Ditch	Nete/Scheldt	Mol	51° 12′ 19.84″ N	5° 12′ 36.43″ E
K1	Kneutersloop	Nete/Scheldt	Olen	51° 10′ 37.70″ N	4° 52′ 03.10″ E
K2	Kneutersloop	Nete/Scheldt	Olen	51° 11′ 14.70″ N	4° 52′ 30.30″ E

Table S3. 28 morphological scoring parameters used in this study.

	Developmental Timepoint (hpf)				
**Morphological endpoint**	24	48	72	96	120
Coagulation	•	•	•	•	•
Tail detachment	•				
Somite formation	•				
Presence of heartbeat	•	•	•	•	•
Hatching		•	•	•	•
Curvature abdomen					•
Curvature tail					•
Elbow tail					•
Tissue deviation tail					•
Oedema head					•
Oedema pericard					•
Oedema yolk					•
Blood accumulation tail					•
Blood accumulation head					•
Blood accumulation heart					•
Blood accumulation yolk					•
Blood accumulation yolk extension					•
Missing left/right pectoral fin					•
Malformation left/right pectoral fin					•
Malformation yolk					•
Malformation heart					•
No or disturbed blood circulation tail					•
Deviating shape of head					•
Malformation ear					•
Malformation eye					•
Malformation mouth					•
Deviating pigmentation					•
Swim bladder inflation					•

Table S4. Overview of different control solutions in field and reconstituted experiments.

Field experiment	Reconstituted experiment
**Control** = standard embryo medium	**Control** = standard embryo medium
**Control D** = RO water with Instant Ocean salts, conductivity and pH adjusted to the field water sample of the ditch	**Control D** = RO water with Na, Ca, K and Mg in the same concentrations as found in the ditch
In the field experiment no additional control solution was used for the SN1 and SN2 sampling points because the pH and conductivity measured in SN1 and SN2 were sufficiently similar to standard embryo medium	**Control SN1** = RO water with Na, Ca, K and Mg in the same concentrations as found in the SN1 sampling point
	**Control SN2** = RO water with Na, Ca, K and Mg in the same concentrations as found in the SN2 sampling point
**Control K** = RO water with Instant Ocean salts, conductivity and pH adjusted to the field water sample of the Kneutersloop	K was not reconstituted since exposure to field samples of K did not cause effects.
